# Intravital Monitoring of Psoriasis Treatment Response and Drug Delivery Using Multiphoton Fluorescence Lifetime Imaging

**DOI:** 10.1111/srt.70367

**Published:** 2026-06-11

**Authors:** Lynhda Nguyen, Christian Mess, Katharina Herberger, Stefan W. Schneider, Volker Huck

**Affiliations:** ^1^ Department of Dermatology and Venereology University Medical Center Hamburg‐Eppendorf Hamburg Germany

**Keywords:** drug delivery, fluorescence lifetime imaging, intravital imaging, multiphoton tomography, psoriasis

## Abstract

**Background:**

Multiphoton tomography equipped with fluorescence lifetime imaging (MPT‐FLIM) is a novel noninvasive imaging technique for analyzing morphological and metabolic states of skin diseases at a subcellular resolution. The present study is the first to establish MPT‐FLIM as an imaging modality to monitor treatment response in psoriasis during topical anti‐inflammatory therapy.

**Materials and Methods:**

Patients with psoriasis treated with topical calcipotriol/ betamethasone dipropionate (C/B) or a calcipotriol derivative (C/‐) formulation were recruited and monitored using MPT‐FLIM. Imaging was performed at baseline on day 0, and during treatment on days 3 and 28.

**Results:**

A total of six patients prescribed C/B, six patients prescribed C/‐ and four healthy controls were recruited. Characteristic histological features were visualized, including acanthosis, parakeratosis, papillomatosis, and thinning of the granular layer. There was a strong correlation between clinical, multiphoton tomographic, and pathophysiological improvement during treatment. Assessment of subclinical metabolic changes was a predictive parameter for treatment outcome. Detection of fluorescence signals from drug components allowed for tracking of drug distribution in intra‐ and intercellular spaces.

**Conclusion:**

MPT‐FLIM proved to be a suitable tool for monitoring treatment response in psoriasis patients and tracking drug delivery. It could be a potential method for monitoring other inflammatory skin diseases during treatment to adjust therapy on an individual level.

## 1 Introduction

Psoriasis vulgaris is a common chronic inflammatory skin disease characterized by sharply demarcated, erythematous‐squamous plaques [1, 2]. Symptoms include itch, pain and bleeding [3]. Due to reported symptoms and striking appearance, psoriasis can cause a psychosocial burden and a massive impact on the health‐related quality of life [4, 5, 6]. A wide range of therapies is available for psoriasis, including topical and systemic treatments. In chronic plaque psoriasis, topical therapy is the cornerstone of treatment. According to the current literature, the combination of a vitamin D analogue and potent corticosteroids is the most effective anti‐inflammatory intervention [7, 8].

Monitoring the course of psoriasis plaques during treatment is essential for evaluating drug efficacy and safety. Physical examination of human skin is limited to the macroscopic surface and the application of validated assessment scales. Here, the Psoriasis Area and Severity Index (PASI) is a common score to quantify the severity of redness, thickness and scaling of psoriasis plaques [9]. For high‐resolution analysis, conventional histology is useful but limited by its invasiveness, potential scarring, and single‐time‐point assessment. To dynamically monitor the course of skin diseases before and during treatment, noninvasive intravital techniques are necessary.

Multiphoton tomography equipped with fluorescence lifetime imaging (MPT‐FLIM) can make a significant contribution to this diagnostic need. By applying near infrared laser technology with femtosecond laser pulses, MPT‐FLIM obtains in vivo images of the skin at subcellular resolution as a proven safe method [10]. Excitation of endogenic fluorophores and second harmonic generation (SHG) provide visualization of cellular and extracellular components, including living cells, mitochondria, the extracellular matrix, and cosmetic and pharmaceutical components [10, 11, 12]. A major component of the intravital FLIM signal is nicotinamide adenine dinucleotide (NADH) [13, 14]. The relationship of free and protein‐bound NADH reflects the current metabolic state of the skin [15].

To our best of knowledge, no studies have been conducted to monitor treated psoriasis vulgaris with MPT‐FLIM to date. This clinical study introduces MPT‐FLIM as an intravital diagnostic tool to assess the morphological and metabolic course of psoriasis and track drug delivery during topical treatment with calcipotriol and betamethasone dipropionate (C/B) or calcipotriol derivative (C/‐).

## 2 Material and Methods

### 2.1 Study Design

This study was designed as an observational, controlled trial. It was approved by the local ethics committee (2020‐10200‐BO‐ff) and conducted to the guidelines of the Declaration of Helsinki and The International Conference on Harmonization of Technical Requirements for Registration of Pharmaceuticals for Human Use (ICH). After informed consent, patients with a clinical diagnosis of psoriasis vulgaris and a planned topical therapy were recruited. They received either a C/B (Enstilar, 0.05 mg/g + 0.5 mg/g, Leo Pharm GmbH) or a C/‐ formulation (Calcipotriol, 0.05 mg/g, Hexal AG) once daily. Additionally, age‐correlated healthy controls were included. Exclusion criteria were topical anti‐inflammatory therapy in the last two weeks and systemic anti‐inflammatory treatment in the last four weeks.

### 2.2 Study Protocol

In each patient, three lesional skin areas were analyzed using MPT‐FLIM at day 0 (baseline) and during topical therapy at day 3 and day 28. Each lesional skin area was monitored performing two independent MPT‐stacks with an vertical image distance of 1.5 µm from the stratum corneum to the papillary dermal layer. At each visit, photographic documentation and clinical assessment using the PASI score were performed by a board‐certified dermatologist. Healthy subjects equally underwent MPT‐FLIM analysis. Any adverse events were documented and treated accordingly. Primary endpoints were (i) intravital morphological and metabolic characterization of psoriasis and (ii) progression of subcellular changes of psoriasis during anti‐inflammatory therapy. Secondary outcome parameter was the MPT‐FLIM‐based drug tracking; therefore, to determine the drug‐related FLIM components, we measured the decay time of the applied topicals by fluorimetric characterization at an excitation wavelength of 380 nm, and MPT‐FLIM measurements were additionally performed two hours after the first topical application in the C/B and C/‐ groups.

### 2.3 Multiphoton Tomography and Fluorescence Lifetime Imaging

In vivo imaging was performed using a CE‐certified multiphoton tomographic system (MPTflex, JenLab GmbH, Berlin, Germany). An excitation wavelength of 760 nm with a pulse duration of 100 fs provided by a titanium:sapphire tunable laser system (Mai Tai, Newport Spectra‐Physics, Santa Clara, CA, USA) was used to excite autofluorophores and to induce SHG. The device was equipped with a Glan calcite polarizer and two galvanometric mirrors. After passing a beam expander and collimator, the excitation laser beam was reflected into a 40x oil immersion objective lens with a numerical aperture of 1.3 (Carl Zeiss Jena GmbH, Jena, Germany) [16, 17]. Further, a short‐ and long‐pass filter set (F75‐680 Multiphoton‐Emitter HC 680/SP and F39‐409 BrightLine HC 409/LP; Semrock Inc., Rochester, NY, USA) and a beam splitter (F25‐660 660DCXR; Chroma Technology Corp., Bellows Falls, VT, USA) for the autofluorescence pathway were installed. A Schott BG 39, a FF01‐380 BrightLine BP 380/14 bandpass filter and a FF409 BS409 beam splitter (all Semrock Inc., Rochester, NY, USA) were utilized for the SHG pathway.

### 2.4 Data Acquisition and Processing

FLIM analysis was performed using SPCImage 8 software (SPCM, Becker&Hickl GmbH, Berlin, Germany). The mean fluorescence lifetime (*τ*_m_) was calculated as an objective parameter of the metabolic state, reflecting the relationship between the fluorescence lifetimes of free (short *τ*_1_) and protein‐bounded (long *τ*_2_) NADH and their ratios a_1_ and a_2_, defined as:

$$\tau m=\frac{a1\tau 1+a2\tau 2}{a1+a2}$$

However, before analyzing the cellular metabolic state of the target areas, the drug‐derived fluorescence signal (*τ*_3_) had to be considered. After fluoremetric characterization of both topical formulations, we therefore used a three‐component fitting approach with a fixed *τ*_3_ and calculated *τ*_m_ using only the first two components *τ*_1_ and *τ*_2_ to get focus on the true metabolism of the tissue. The fluorescence lifetimes were pseudo‐color coded ranging from 100 ps (red) to 2000 ps (blue).

### 2.5 Statistical Analysis

Statistical analysis was performed using SPSS software (Version 28, IBM Company). Since the Shapiro‐Wilk tests were positive as expected in the relatively small samples of our collected data set, nonparametric Kruskal‐Wallis analysis of variance (ANOVA) was used to determine the difference in mean between groups. Equivalence testing and stated effect sizes were performed using G*Power (Version 3.1.9.7, University Kiel, Germany). *P*‐values < 0.05 were considered significant. Means are presented with standard errors of the mean (SEM) and ranges.

## 3 Results

### 3.1 Basic Characteristics

In total, six patients with prescription for C/B, six patients with C/‐ and four healthy controls were recruited. Of the twelve patients initially enrolled, ten completed the entire trial. Two patients with C/B were excluded due to systemic glucocorticoid treatment and nonresponse to the anti‐inflammatory treatment within the study interval. A total of 156 optical biopsies were performed. Subject demographics are shown in **Table** **1**.

**TABLE 1:** Subject demographics. C/B: Calcipotriol/ betamethasone dipropionate combination. C/‐: Calcipotriol derivate.

Patient group	Gender [n female/male]	Age [mean years ± SEM]	Fitzpatrick skin type [*n*]	PASI score at baseline
Patients treated with C/B	2/4	51 ± 2.6	II: 4 III: 2	7 ± 0.8
Patients treated with C/‐	2/4	50 ± 3.1	II: 4 III: 2	6 ± 0.6
Healthy controls	1/3	50 ± 2.2	II: 3 III: 1	Not applicable

### 3.2 Macroscopic Assessment

After three days of C/B treatment, a positive therapeutical response was documented as reflected in the PASI score: The mean percentage change was 9.0 % after 3 days, and 78.2 % after 28 days. In the patient group treated with C/‐, the mean percentage PASI score change score was 2.4 % after day 3, and 72.2 % after day 28.

### 3.3 Multiphoton Characterization of Treated Psoriasis

**FIGURE 1:**
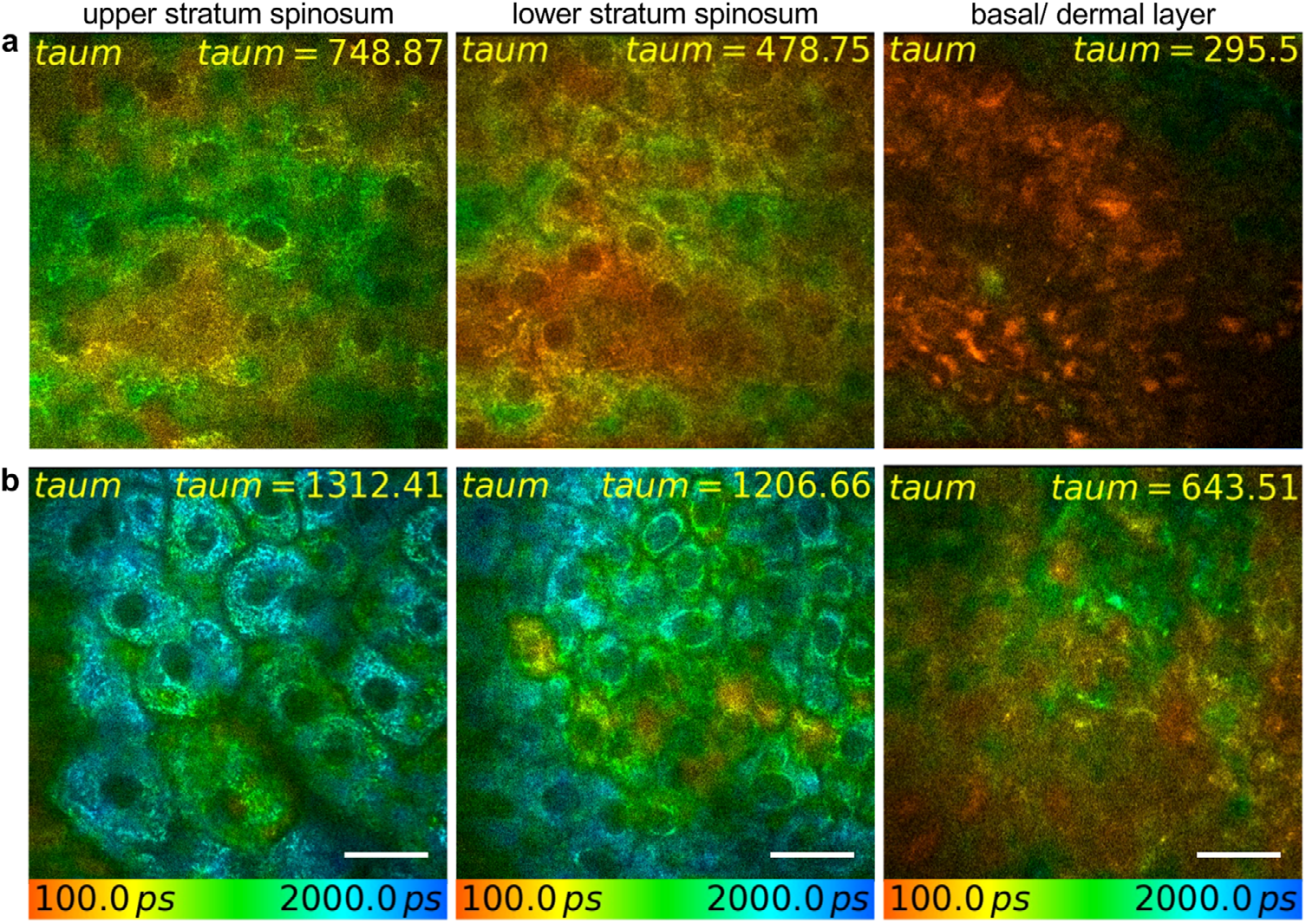
Morphological and metabolic alterations of psoriasis plaques compared to healthy skin were examined using multiphoton tomography and fluorescence lifetime imaging (MPT‐FLIM). (a) Psoriasis patients exhibited enlarged and irregular intercellular spaces, alongside irregularly shaped keratinocytes with a higher nucleus to cytoplasm ratio compared to healthy controls. The presence of inflammatory edema and hyperkeratosis resulted in laser scattering, limiting visualization of deeper structures. (b) In contrast, healthy skin showed regularly shaped keratinocytes with uniform intercellular spaces. Notably, the mean fluorescence lifetime (*τ*m) in psoriasis plaques was significantly lower than in healthy skin indicating the chronic inflammatory state. Scale bar: 20 µm.

By detecting autofluorescence signals and SHG, characteristic histological features of psoriatic plaques could be visualized, including acanthosis and parakeratosis (**figure** **1**). Underneath the stratum corneum, a heterogeneous punctiform fluorescence pattern was seen, in agreement with previous studies [18]. This pattern, which does not correlate with the corneal or granular layers, is assumed to be the result of incomplete differentiation of keratinocytes [18]. The granular layer appeared to be reduced and, in some cases, absent. Dermal papillae infiltrated into the epidermal layers. Psoriatic lesions showed a higher ratio of nucleus to cytoplasm compared to healthy subjects. During C/B and C/‐ treatment, cellular atypia was reduced but with ongoing minimal acanthosis of psoriasis despite clinical clearance of lesions on day 28. Healthy controls showed no significant changes during the interval observation.

### 3.4 Fluorescence Lifetime Imaging of Drugs

**FIGURE 2:**
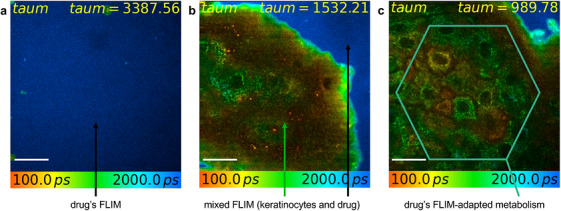
Multiphoton tomography and fluorescence lifetime imaging of psoriasis plaques two h after calcipotriol/betamethasone dipropionate application. (a) shows the target area directly above the stratum corneum. (b) visualizes cells in the upper stratum spinosum. (c) addresses central stratum spinosum. The wrinkle in the upper right corner seems to be filled with drug's FLIM signal. The different FLIM components (metabolism‐ and drug‐related) are depicted as indicated and marked by the arrows. Scale bar: 20 µm.

We measured an isolated fluorescence decay time of around 4000 ps in both topical formulations (*τ*_3_). Although morphologically indistinguishable, the fluorescence lifetimes of the C/B and C/‐ formulations differ significantly from the intended NADH measurements. Early after the first application, the signals of *τ*_m_ and *τ*_3_ were still separated. At subsequent visits, an overlap was observed, indicating a cellular uptake. **Figure** **2** shows the overlapping drug‐related FLIM components and illustrates the need for FLIM adaption. By detecting drug's specific fluorescence, autofluorescence signals of skin and drug components could be distinguished. A few hours after first application, diffusion of C/B and C/‐ formulations from the epidermal layer into the upper dermis could be visualized. In addition, topical formulation appeared to accumulate in wrinkles which may act as a storage depot (**figure 2c**). Intracellular accumulation could be discriminated computationally from autofluorophores and visualised by pseudo‐color coding in bright blue.

### 3.5 Drug's FLIM‐Adapted Metabolism

**FIGURE 3:**
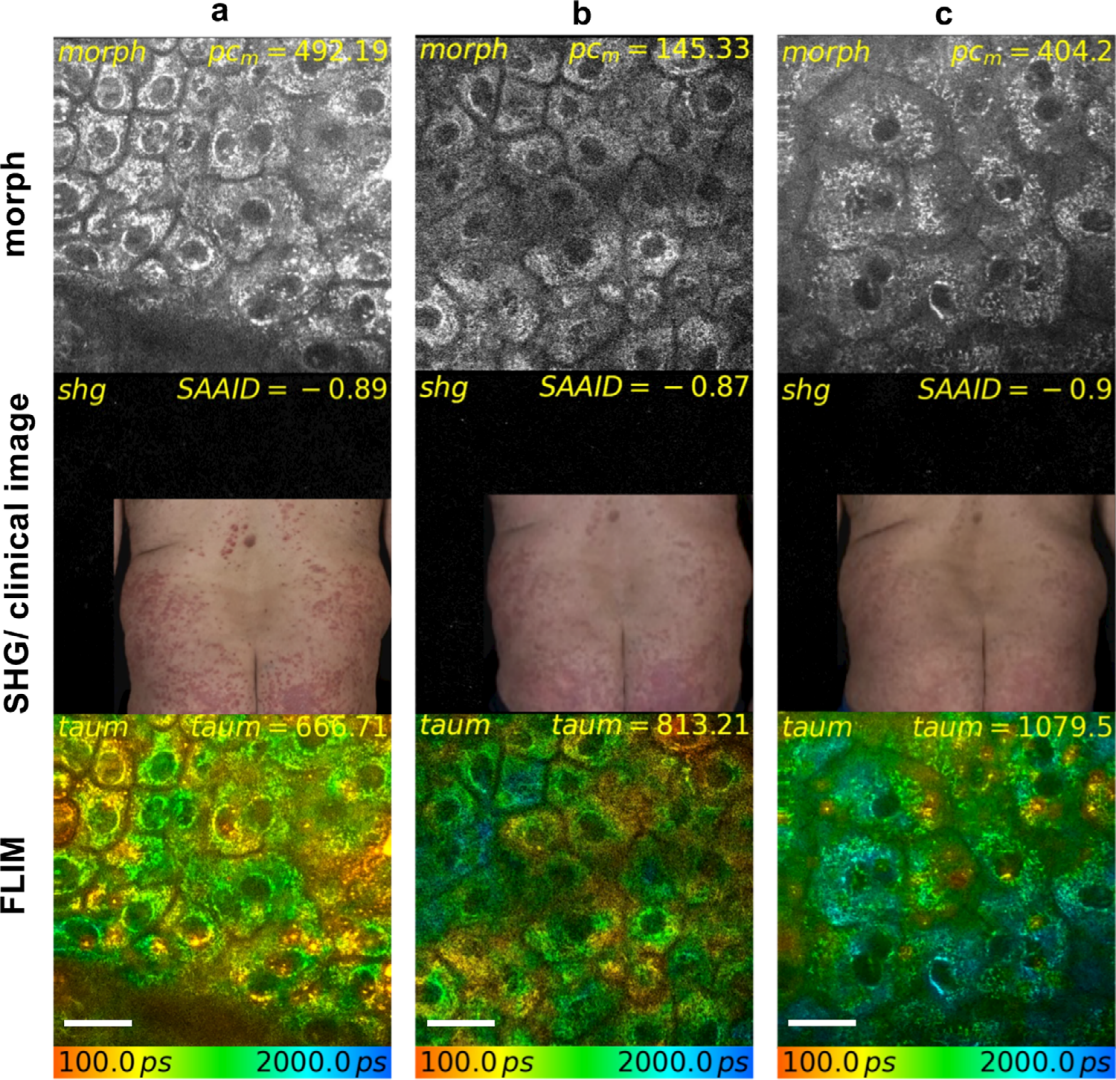
Multiphoton tomography and fluorescence lifetime imaging (MPT‐FLIM) of one exemplary patient who underwent full clearance after 28 days of topical calcipotriol/betamethasone dipropionate treatment. Measurements at (a) day 1, (b) day 3, (c) and day 28 were conducted. A normalisation of drug's FLIM‐adapted metabolism in the upper stratum spinosum could be detected (bottom row). Scale bar: 20 µm. morph: morphology. SHG: second harmonic generation. FLIM: fluorescence lifetime imaging.

After FLIM adaptation of patients treated with C/B and C/‐, the course of the cellular metabolic state of the upper stratum spinosum measured by MPT‐FLIM was consistent with macroscopic observations. On day 0, FLIM analysis revealed a significant difference in mean *τ*_m_ (751 ± 120 ps) compared to healthy control (1100 ± 77 ps), indicating a chronic inflammatory state (*p* < 0.05, d = 3.462). During topical C/B treatment, *τ*_m_ increased notably on day 3 (802 ± 90 ps) and reached 1001 ± 98 ps at day 28. In patients treated with C/‐, *τ*_m_ also increased to 951 ± 121 ps (day 28) but significantly differed from the *τ*_m_ value of C/B patients and healthy controls (*p* < 0.05). *τ*_m_ of healthy controls showed no difference during the study period (**Table** **2**). **Figure** **3** shows 3‐component‐fitting FLIM measurements in a patient who experienced lesion clearance under C/B treatment.

**TABLE 2:** Summary of mean fluorescence lifetimes of healthy subjects and psoriasis patients treated with calcipotriol/ betamethasone dipropionate (C/B) or a calcipotriol derivative (C/‐) formulation over time. Effect size *d* as stated, *p*‐values < 0.05 were considered statistically significant.

Timepoint of measurement	Healthy control	Psoriasis vulgaris treated with C/B	Psoriasis vulgaris treated with C/‐
Day 0	1100 ± 77 ps	751 ± 120 ps (*p* < 0.05, *d* = 3.462)	
Day 3	1113 ± 47 ps	802 ± 90 ps (*p* < 0.05, *d* = 4.332)	775 ± 45 ps (*p* < 0.05, *d* = 7.346)
Day 28	1075 ± 97 ps	1001 ± 98 ps (*p* < 0.05, *d* = 0.759)	951 ± 121 ps (*p* < 0.05), *d* = 1.131)

## 4 Discussion

The present study provides the first evidence for intravital high‐resolution monitoring of psoriasis vulgaris during topical anti‐inflammatory therapy and drug delivery using MPT‐FLIM. Through a controlled, observational study during C/B and C/‐ treatment of psoriasis patients, we identified a high correlation between clinical, multiphoton tomographic, and metabolic improvements.

MPT‐FLIM derived metabolic characterization is achieved by analyzing and quantifying the relationships between short fluorescence lifetimes of 200 to 450 ps, representing free NADH, and longer lifetimes of 2000–3000 ps, indicating protein‐bound intracellular NADH. Hereby, we obtained a direct insight into the metabolic state of a single cell. To condense: Is NADH mainly bound to the inner mitochondrial membrane in the process of oxidative phosphorylation or is the free NADH predominant indicating the metabolic process of glycolysis? Before treatment, characteristic microscopic changes of psoriasis plaques were easily visualized using MPT‐FLIM. In addition, we were able to show that the individual course of lesion healing correlated with cellular morphologic and metabolic state in the upper stratum spinosum in accordance with the PASI scores. A valid analysis of the metabolic status using a 3‐component fitting algorithm could only be achieved by post hoc arithmetic removal of the drug‐induced FLIM signal. Although patients experienced complete clearance of psoriatic plaques at the last visit, MPT‐FLIM still detected subclinical morphological and pathophysiological signs of inflammation. Identification of these areas will be beneficial in initiating proactive treatment and preventing exacerbation of skin lesions.

To date, only few studies have dynamically monitored psoriasis lesions under therapy using noninvasive intravital tools, mostly applying reflectance confocal microscopy (RCM) [19, 20, 21]. RCM has been used to visualize characteristic histological features of psoriasis. Our results confirm previous findings on MPT‐FLIM as a noninvasive intravital imaging tool for monitoring the course of skin diseases [10, 22, 23]. To date, to the best of our knowledge, no studies have been published on MPT‐FLIM as a tool for monitoring treatment response in psoriasis. Few studies have shown that MPT‐FLIM is suitable for imaging microscopic features of psoriasis [18, 24]. FLIM has been proven to be useful for imaging autofluorophores, e.g. FAD, free and bound NADH. As these molecules are fundamental to metabolism, MPT‐FLIM could make an important contribution to the present diagnostic gap. MPT‐FLIM has also been applied to other inflammatory skin diseases, such as atopic dermatitis, as well as various types of skin cancer, demonstrating promising potential as a diagnostic tool [25, 26]. However, due to its novelty in imaging tools, further research is necessary. We hypothesize that MPT‐FLIM could serve as a valuable tool for distinguishing mimickers in a manner similar to histological morphological examination. Given its ability to provide noninvasive, high‐resolution imaging at the cellular and metabolic levels, MPT‐FLIM holds potential for enhancing the early detection and differentiation of skin lesions. To validate this hypothesis, further studies are necessary, incorporating classical histological analysis as a reference standard for comparison.

First described by König et al., femtosecond laser imaging systems enable a unique high‐resolution visualization of pharmaceutical and cosmetic components in their natural environment [11]. In agreement with the authors, we were able to demonstrate the capabilities of MPT‐FLIM for advanced drug tracking. By detecting drug‐specific fluorescence signals, in vivo intra‐ and intercellular distribution and accumulation of drug components could be tracked while maintaining discrimination from individual skin autofluorescence signals. Additionally, few studies have investigated the long‐term use of MPT‐FLIM in monitoring newly injected tattoos and its role in laser‐assisted delivery of hyaluronic acid in human skin [12, 27, 28]. They have demonstrated that MPT‐FLIM can effectively track the biodistribution of external products within the skin, and its impact on the metabolic status of surrounding tissues over several weeks. Previous studies mainly applied confocal laser scanning microscopy or optical coherence tomography for intravital drug screening [29, 30, 31]. These imaging tools provide a sufficient overview of the upper skin layers but are limited by phototoxicity and low resolution, resulting in loss of information. MPT‐FLIM could facilitate local drug tracking and simultaneous assessment of pharmacokinetics and cellular and physiological response to treatment.

The ability of MPT‐FLIM to visualize subclinical changes allowed early assessment of treatment response. For example, we observed one subject in the C/B group who did not respond adequately to the treatment and therefore dropped out of the study. Psoriasis lesions were still present and PASI score—expecting a “PASI 25”, an intended therapeutic improvement of at least 25% decrease of the initial score value—was not reduced over a period of 21 days. On day 3, drug‐adapted mean *τ*_m_ showed no significant change (739 ps versus 723 ps at baseline visit). On the drop out day 21, *τ*_m_ minimally increased to 780 ps indicating low to missing treatment response. As patient adherence to treatment is crucial for the desired outcome in psoriasis, early assessment of response is needed. Here, MPT‐FLIM may provide predictive parameters of treatment outcome before macroscopic changes are visible. By detecting non responders to topical treatments at early stage, individual therapy adjustments can be provided and thus, treatment and patient compliance can be managed and optimized more efficiently.

There are some limitations to this study. Due to the small sample size, a bias in the results may be possible. In addition, we have waived adjustments for multiple comparisons not to potentially obscure purposeful findings. While this approach can be justified in exploratory research, it increases the risk of false positives errors. The version of the MPT‐FLIM device that we used had some limitations in handling and therefore limitations in clinical use. Certain areas, such as the scalp, hands, and regions over joints, presented particular challenges for accurate measurement. In addition to the maximum penetration depth of approximately 200 µm, the hyperkeratosis in psoriasis may result in photon scattering or absorption, potentially leading to limited visualization of deeper structures. Due to the observational design of the study, a standardized dosage per surface area was not monitored. As a result, topical formulations were prescribed by the patients’ treating dermatologists rather than dispensed in uniform quantities by the study team. This design‐related limitation may have resulted in variability in application.

Moreover, MPT offers only a limited view of areal lesions, necessitating spot measurements for larger plaques. In its current state, MPT requires a high level of expertise in handling and interpreting the data.

## 5 Conclusion

MPT‐FLIM is a suitable tool to dynamically monitor the therapeutic response of chronic psoriatic plaques. By visualizing endogenous fluorophores and SHG signals, the morphological and metabolic state of the tissue can be assessed during treatment. FLIM of pharmaceuticals can be used for computational screening and tracking of drug delivery. In addition, MPT‐FLIM may have the potential as a predictive tool for treatment outcome at early stage. Further prospective clinical trials are required to evaluate the application of MPT‐FLIM to the treatment of other inflammatory skin lesions and its potential in drug delivery research.

## Ethics Statement

This study was approved by the local ethics committee (2020‐10200‐BO‐ff). Written consent was obtained from the patient for the publication of the images.

## Conflicts of Interest

Volker Huck and Stefan W. Schneider received research fees in terms of an investigator initiated trial by Leo Pharma GmbH. Lynhda Nguyen, Christian Mess and Katharina Herberger have no conflicts of interest to be declared.

## Data Availability

The data that support the findings of this study are available on request from the corresponding author.
